# Retrospective Analysis of Heartworm (*Dirofilia immitis*) Prevention Medication Compliance and Economic Value in Dogs in Veterinary Practices in Australia

**DOI:** 10.3389/fvets.2020.602907

**Published:** 2021-01-05

**Authors:** Kennedy Mwacalimba, Andrea Wright, Konstantinos Giannakakis, Richard L'Estrange, Tinh-Son Nguyen

**Affiliations:** ^1^Outcomes Research, Zoetis, Parsippany, NJ, United States; ^2^Athens Technology Center S.A., Halandri, Greece; ^3^Zoetis Australia, Silverwater, NSW, Australia

**Keywords:** heartworm, dog, extended-release, injectable moxidectin, revenue, *Dirofilaria immitis*

## Abstract

**Background:** Canine heartworm (HW) is endemic in Australia. Prevention usually involves monthly topical or oral preventives, or annual injections of extended-release moxidectin (ProHeart SR-12^*^^1^), hereafter referred to as injectable moxidectin (IM). Poor compliance can leave dogs susceptible to infection. This pharmacoeconomics study used retrospective transactional data from 52 Australian veterinary practices to examine the economic value of compliance, revenue, and patient retention associated with veterinarian-sourced canine HW prevention.

**Methods:** This longitudinal descriptive study utilized anonymized transaction records of 228,185 dogs identified to have visited a veterinary practice at least twice in the period 2010–2015. Purchase compliance against a benchmark of 12 months HW protection per year was measured for IM or monthly HW (MHW) preparations each year and for consecutive years. The average annual cost per dog by preventative modality was also determined.

**Results:** Between 2010 and 2015, of the 228,185 dogs identified, 73.0% recorded either zero or one purchase of HW preventive from their veterinary clinic; 18.7% recorded at least two IM purchases, and 10.6% purchased MHW prevention at least twice. Single-year purchase compliance was 92.8–96.9% for IM vs. 26.9–36.5% for dogs receiving MHW products. Consecutive-year purchase compliance was 76.7% for IM and 24.4% for MHW medications. Dog owners spent $AU108.29/dog/year (Australian dollars) on IM vs. $AU131.96/dog/year on MHW prevention products, which may have treated other parasites concurrently, although repeat MHW purchasers only purchased enough to cover an average of 7.2 months per year. Dogs recording at least two HW prevention transactions generated more revenue for veterinary practices/dog/year compared to dogs with less than two. Finally, dogs receiving IM, especially those that started at <15 months old, had the highest retention rate in this population.

**Conclusions:** In the 5 years from 2010 to 2015, 73% of dog owners who visited a veterinary practice at least twice made less than two purchases of HW preventatives from the veterinary practice. For those with at least two preventative purchases, 76.7% of dogs receiving IM and 24.4% of dogs prescribed with MHW products purchased enough doses to provide continuous protection over the observation period.

## Background

In an environment where veterinarians should be playing a stronger role in recommending canine heartworm disease (HW) prevention, there is a paucity of data on how HW preventive compliance impacts practice revenue and client retention over time. Canine heartworm (HW) is found worldwide and is endemic in many countries (1). For example, in the United States (US), HW is endemic in each of the contiguous 48 states, Hawaii, Puerto Rico, US Virgin Islands, and Guam (2). HW is also endemic in many European countries (3) and in Australia (4). HW is caused by the parasitic nematode *Dirofilaria immitis*. Transmission occurs when competent mosquito vectors ingest *D. immitis* microfilariae from an infected host and then transmit infective L3 larvae when they feed on a susceptible dog (5). As larvae mature and migrate within the tissues of the recipient dog, eventually reaching the pulmonary arteries and the heart, infection can cause severe and potentially fatal disease (2, 4). Even low worm burdens can produce profound pulmonary vascular and parenchymal disease, especially in smaller breed dogs (6).

Despite improvements in diagnostic capability and increased availability of preventative products, HW is becoming more prevalent, including in areas of the world previously deemed low risk (6). For example, a 2016 American Heartworm Society (AHS) survey showed a 21.7% increase in the average number of dogs diagnosed positive for adult HW per clinic between 2013 and 2016 (7). Moreover, a European survey of veterinary practitioners found that more than one third believed that HW infection could become endemic in non-endemic areas within the next 10 years (8). The prevention approach for HW disease in dogs is based almost entirely on the administration of one of several macrocyclic lactones, which can be administered monthly as topical or oral preparations, or semiannually (outside Australia) or annually in some countries, including Australia, as injections of the macrocyclic lactone extended-release moxidectin [injectable moxidectin (IM)] (9). Macrocyclic lactones in monthly formulations are approved to eliminate larval stages that are up to 30 days old (9, 10). In Australia, IM (ProHeart SR-12, Zoetis) has a reach-back label claim for the elimination of immature larval stages up to 90 days old when administered at the recommended dose, in addition to preventing HW infection for at least 12 months beyond the date of injection and controlling adult and immature canine hookworm species both at the time of injection and for 4 months beyond (11). The AHS guidelines state that dogs should be on approved HW preventive year-round (2). The Australian Heartworm Advisory Panel also has guidelines that state that all domestic dogs should be on year-round HW prevention irrespective of their location in Australia (12).

Compliance is a primary focus in HW prevention since it has been shown that most dogs that become HW-positive have received no HW prevention or have experienced a gap in HW protection due to non-compliance (13). While resistance to macrocyclic lactones in *D. immitis* is being observed in the US, raising concerns of apparent loss of efficacy of this drug class for canine HW prevention (14), it seems likely that lack of compliance is still the main reason why dogs receiving HW preventive medication are becoming HW-positive.

In Australia, the prevalence of canine HW, while generally considered low by veterinary practitioners, remains poorly understood. In contrast, in the US, a series of monitoring mechanisms have been established. The AHS collects data on HW testing every 3 years (15), and the Companion Animal Parasite Council collects it every month (16). The US also has the Environmental Protection Agency mosquito control program (17), which looks at vectors for HW. These measures do not exist in Australia; however, there is a voluntary online reporting system—the “HW surveillance project” (18)—and since 2014, more than 2000 cases have been reported, although this is likely an underestimation of positive diagnoses in practices. Nevertheless, over the last 5 years, HW disease has been detected in every Australian state, and microfilaremic dogs act as local reservoirs, which increases the possibility of disease transmission to dogs that have missed doses of monthly HW prevention. Indeed, in a recent Australian study in dogs from Queensland and New South Wales, a large proportion of dogs with microfilaremia were reportedly on rigorous monthly HW prevention (4). In Australia, IM is a once-a-year product for the prevention of HW disease in dogs and is only available through veterinarians (19), whereas monthly products are sold widely without a prescription. Therefore, a large proportion of Australian dog owners source their monthly HW preventatives outside the veterinary clinic, and veterinarians cannot fully track purchase compliance with such products, or whether they are administered at recommended intervals. In summary, a large population of Australian pets remain inadequately protected and are therefore susceptible to infection (4). Poor compliance in Australia is presumed to be due to the availability of HW preventatives without prescription, but in the US, such products are sold only with a veterinary prescription and still show a low rate of compliance. The 2016 AHS survey found that ~2/3 of dogs in the US received no HW prevention each year (20), and a 2016 US American Pet Products Association Survey estimated that only 42% of dog owners in the US gave HW medication to their dog (21, 22).

Pharmacoeconomic compliance studies can aid in understanding how medication compliance varies among patients and how that variation could impact health outcomes (23). The objective of this pharmacoeconomics study was therefore to compare annual purchase compliance between dogs on IM and dogs receiving monthly oral HW preventatives through the veterinary channel in Australia. The current study used retrospective anonymized transactional data from Australian veterinary practices to examine HW prevention measures in dogs in terms of compliance and economic value from a practice perspective.

## Methods

This pharmacoeconomics study aimed to adhere to the guidelines and checklist for a systematic approach to compliance and persistence studies using retrospective databases as published by Peterson et al. (23).

### Study Population

Fifty-two general practice veterinary clinics in Australia whose practice management software programs allowed comparative analysis across the period 2010–2015 participated in the study. The number of clinics per state was approximately weighted to represent the number of practitioners in each state (24) as follows: Australian Capital Territory/Northern Territory/South Australia/Tasmania, six practices combined; New South Wales, 16 practices; Queensland, 12 practices; Victoria, 13 practices; Western Australia, five practices.

### Group Selection Criteria

Inclusion criteria for IM or monthly heartworm (MHW) groups: dogs that had visited the practice at least twice and recorded transactions for at least two injections of IM or at least two purchases of MHW prevention product over the period 2010–2015. Reference group criteria: all dogs that had visited the practice at least twice but recorded zero or one HW prevention product purchase in the period 2010–2015. These dogs were considered not consistently receiving an HW preventive from the practice. Each dog had a distinct animal identification and was only counted once.

### Description of Data

This longitudinal descriptive study utilized historical data from veterinary clinics of anonymized transaction records of products and services purchased by clients for the period 2010–2015. This analysis uses Australian dollars. Data were collected from practice management software in the form of Microsoft Excel files. The records comprised 5,139,337 rows of data from 1,951,652 individual invoices. These represented total purchases of $AU 264,473,786 (US$190,421,126) for 228,185 individual dogs.

Each spreadsheet contained the following: unique animal identification number; animal name; date of birth; canine species; animal weight; consult date; item code; item name; item sales price; and item service sales price. Exclusion criteria were as follows: invoices for non-canine species and invoices with missing data in any of the following columns—animal identification, product description, price, and invoice date.

In order to correctly classify dogs as IM dogs or MHW prevention group dogs, a list of all possible products available in Australia was compiled for these two product categories, and product name permutations for each were identified in each clinic's transaction records. Through this process, product keys were created and refined by hand for IM or MHW prevention products to enable data queries and perform product-based calculations. MHW products contain differing numbers of doses, with most product descriptions containing a reference to the number of doses (e.g., 3 pack, 6s, etc.). By applying a proper text matching process, we were able to identify the number of doses for these product purchases. For product descriptions with no packaging information, the number of doses was estimated using a decision tree, which utilized the average price and the average weight of the dogs for which purchases were made.

### Variables Analyzed

#### Purchase Compliance

Looking only at dogs that had two or more HW transactions during the observation period, the following definitions were used:

- Single-year purchase compliance (SYPC):
  For the IM group: For dogs over 15 months of age, purchase of IM in the course of a year, with at least two injections over the study period, regardless of the interval between injections, was classified as compliant. For dogs under 15 months of age, purchase of at least two IM injections since birth was required for single-year compliance (Note that, in Australia, IM is registered for use in dogs from 12 weeks of age. Because puppies are growing, the protection given at this young age may not last 12 months. Continuous protection requires repeated injections, and a dog receiving ProHeart SR-12 that is younger than 15 months should have received at least two injections by the time it was 15 months of age. One of the most used protocols is the following: first injection at 12 weeks of age—second injection at 6 months of age and third injection at 15 months of age. Each injection coincides with another routine intervention−12 weeks: second puppy vaccination−6 months: neutering time−15 months of age: first yearly vaccine booster) For MHW recipients: An MHW dog younger than 12 months was considered to be compliant if as many doses were purchased as its age in months at the end of the year. For older dogs, 12 monthly doses were required.
- Consecutive-year purchase compliance (CYPC): Dogs had to be single-year purchase compliant for at least two consecutive years to be considered consecutive-year purchase compliant.
- Non-compliant reference group: Dogs recording either zero or one purchase of HW preventive from their veterinary clinic over the observation period.

### Revenue

The total annual revenue per dog from each of the three groups (IM, MHW, and reference groups) was calculated by adding all the invoices (for all products and services) for each year for each group and dividing by the number of dogs in each group for that year.

### Retention

Clinic retention was plotted over the years of the observation period for the different age categories of dogs in the IM group vs. all dogs in the study population. All dogs that got their first injection of moxidectin were identified in a specific year, and transactions were followed for all subsequent years to find out whether they kept returning to the practice. The plot could not identify the reasons for failure to return, which could have included mortality or moving to a different practice.

### Costs of HW Prevention

The average annual cost of HW prevention per dog by preventative modality was determined for all dogs that were single-year purchase compliant. The average annual cost of MHW prevention was calculated as follows: for each MHW invoice, the quantity sold was multiplied by the number of months the product was indicated to prevent HW disease to obtain the total number of months of coverage. Next, the total revenue generated by MHW prevention purchases was divided by the total number of months of coverage sold to obtain the average monthly cost. Finally, this cost was multiplied by 12 to obtain the average annual cost.

### Statistical Analysis

Descriptive statistics of the data are provided. Calculations included determining binomial proportion confidence intervals (CIs; at 95% confidence). CIs for proportions were calculated with R™'s binom package using the asymptotic method at 95% confidence level. CIs for mean values were calculated from t distribution at 95% confidence level.

## Results

### HW Preventative Use

A total of 228,185 dogs were identified that visited the 52 practices at least twice in the period 2010–2015. Of these, 73.0% (*n* = 166,596/228,185) were dogs with less than two HW preventive transactions over that 5-year time period (reference group). For dogs whose owners purchased HW prevention at least twice from their veterinarian, 18.7% (*n* = 42,663/228,185) had purchased IM and 10.6% (*n* = 20,352/228,185) purchased an MHW preventive. There were 1,426 dogs identified with at least two purchases of both IM and monthly preventive.

**Figure 1:**
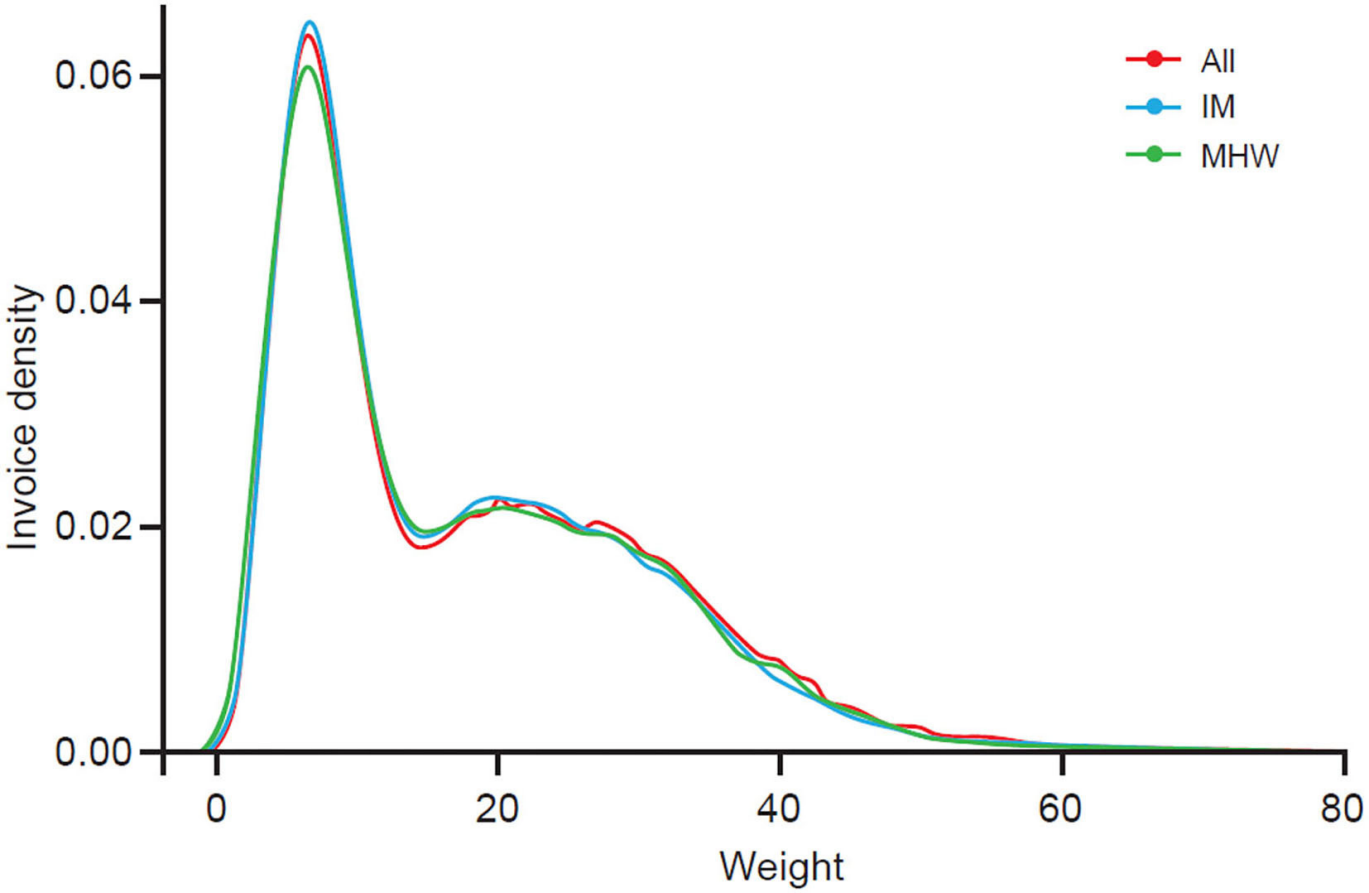
Weight density.

A dog weight density graph for all IM and MHW purchases was created by categorizing weights in bands on the x-axis (e.g., weights from 1.0 up to 1.2 kg were grouped into a band) and counting all purchases that fell into each band. Because the frequencies of dogs not consistently obtaining HW prevention from their veterinarian were higher than the frequencies of the IM or MHW categories, the data were normalized by dividing all frequencies with the total number of category invoices to calculate the density. The density was the highest at the weights that occurred most often in the invoice data. There were no weight (Figure 1) or age differences (data not shown) in the profile of dogs in the three categories evaluated.

### Purchase Compliance

#### Single-Year Purchase Compliance (SYPC)

**Figure 2:**
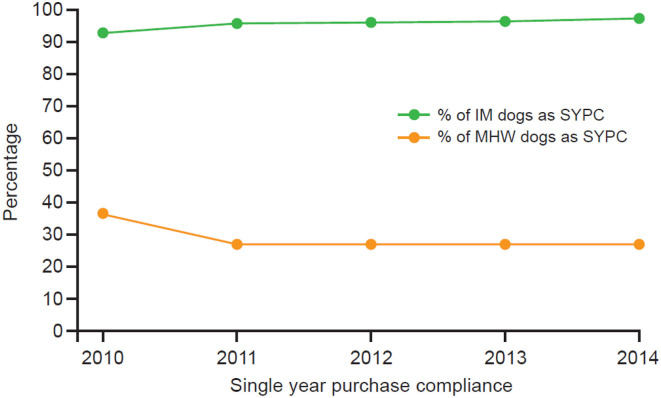
SYPC each year for dogs IM group vs. MHW group.

For the observation period, the mean SYPC for dogs treated with IM was 96.3% (CI = 96.2–96.4%). In comparison, the mean SYPC for dogs receiving MHW products was 28.7% (CI = 28.3–29.1%) (Table 1, Figure 2). Note that the mean SYPC for the IM group is not 100% because dogs under 15 months of age require at least two doses to qualify as compliant. For SYPC of IM, the only means of purchasing IM in any given year and not being compliant was to be a dog under 15 months of age that had not purchased two doses. For MHW dogs, 12 doses of MHW product had to be purchased in the year. For dogs less than a year of age, there had to be an equal number of doses purchased as the dog's age in months at the end of the year to be compliant.

**Table 1:** Comparison of calculated percentage of IM dogs being SYPC compared to MHW prevention dogs being SYPC.

**Year**	**IM SYPC #Dogs**	**IM Non-SYPC #Dogs**	**% of IM Dogs as SYPC**	**MHW SYPC #Dogs**	**MHW Non-SYPC #Dogs**	**% of MHW Dogs as SYPC**
2010	16,025	1,241	92.8%	2,871	4,992	36.5%
2011	20,887	1,018	95.4%	2,217	5,937	27.2%
2012	22,636	969	95.9%	2,141	5,804	26.9%
2013	23,572	941	96.2%	2,202	5,945	27.0%
2014	24,033	760	96.9%	2,134	5,692	27.3%

*IM, injectable moxidectin; MHW, monthly heartworm; SYPC, single year purchase compliant; #number; %percentage*.

#### Consecutive-Year Purchase Compliance (CYPC)

**Figure 3:**
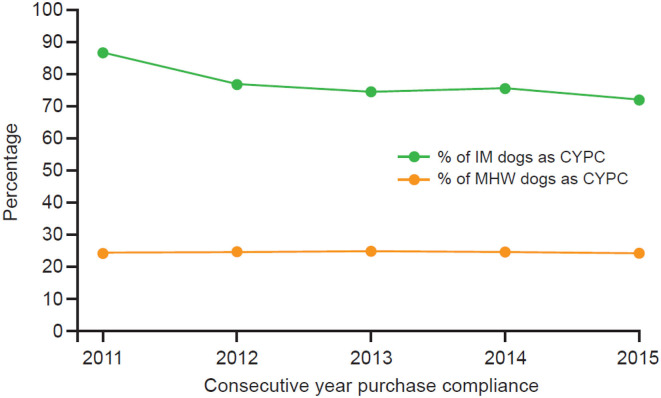
Comparison of CYPC between dogs receiving at least 24 months of continuous protection with IM or MHW prevention. IM, injectable moxidectin; MHW, monthly heartworm; SD, standard deviation.

CYPC is summarized in Table 2, Figure 3. This was calculated using SYPC from the current year and comparing to the SYPC of the previous year. If a dog was SYPC for the current year *and* the previous year, it was considered CYPC. If a dog was SYPC for the previous year but *not* the current year, it was non-CYPC for the current year. The same calculations are performed for consecutive years of purchase for MHW dogs; however, the SYPC dogs from the previous year that did not maintain SYPC for the current year were added to the non-SYPC dogs for the current year to give a total number of non-CYPC dogs. The average CYPC for the IM group over the entire observation period was 76.7% (95% CI = 76.5–76.9%) and for the MHW group 24.4% (95% CI = 24.0–24.8%). Therefore, only 24.4% of dogs on MHW prevention were calculated to have purchased enough products through their veterinarian to provide at least 24 months of continuous protection over the observation period. In comparison 76.7% of dogs receiving a moxidectin injection were assessed as having at least 24 months of continuous protection over the 5-year observation period.

**Table 2:** Comparison of calculated percentage of IM dogs being CYPC compared to MHW prevention dogs being CYPC.

**Year**	**IM SYPC #Dogs**	**#SYPC dogs from previous year not SYPC in current year**	**% of IM dogs as CYPC**	**MHW SYPC #Dogs**	**#SYPC dogs from previous year not SYPC in current year + #Non-SYPC dogs from current year**	**% of MHW dogs as CYPC**
2011	20,887	3,223	86.6%	2,217	6,975	24.1%
2012	22,636	6,825	76.8%	2,141	6,630	24.4%
2013	23,572	7,987	74.7%	2,202	6,711	24.7%
2014	24,033	7,855	75.4%	2,134	6,552	24.6%
2015	20,364	7,926	72.0%	1,725	5,421	24.1%

*IM, injectable moxidectin; MHW, monthly heartworm; SYPC, single year purchase compliant; CYPC, consecutive year purchase compliant; #number; %percentage*.

### Cost of HW Prevention

**Figure 4:**
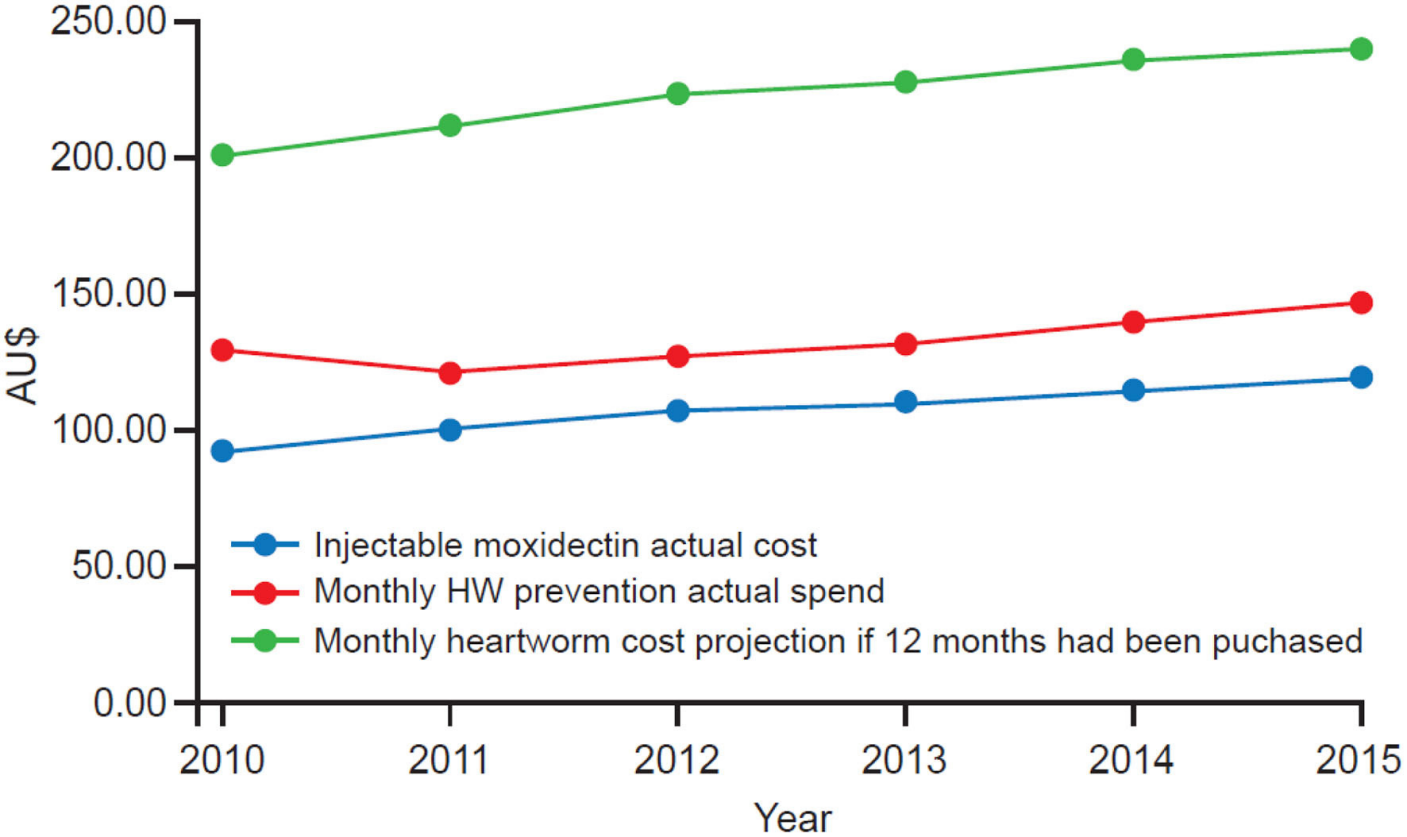
Comparison of the average cost ($AU) of IM and the calculated cost of MHW for 12 months coverage. Figures represent the mean cost per dog.

Dog owners spent a mean of $AU108.29/dog/year (US$77.97 using a November 2020 conversion rate of US$0.72 to $AU1.00) on IM compared to $AU131.96/dog/year (US$95.01) on products approved for MHW prevention. Despite buying fewer months of protection, MHW prevention products cost pet owners more than IM. It should be stated, however, that several monthly products are labeled for more than just HW prevention, providing protection against other parasites, which is reflected in the relative cost. Furthermore, while 24.4% of MHW group dogs were recorded as consecutive-year purchase compliant, the MHW group in entirety purchased only enough products from their veterinarian on average to cover 7.2 months of the year (range 6.9–7.7 months between 2010 and 2015). In contrast, administration of IM provides 12 months of continuous HW protection. The calculated cost of a full 12 months of MHW prevention was $AU221.79/dog/year (US$159.69), i.e., $AU113.50 (US$81.72) more than IM for 12 months of coverage. The average cost per dog of 12 months of MHW doses and the average cost of IM are summarized in Table 3, Figure 4.

**Table 3:** Comparison of the average cost ($AU) of MHW doses (actual spend and projected cost if 12 months had been purchased) and the average cost of IM per dog.

**Year**	**IM average cost**	**SD**	**MHW prevention average actually spent**	**SD**	**MHW cost projection if 12 months had been purchased**	**SD**
2010	$92.72	$36.42	$129.52	$90.82	$200.96	$74.52
2011	$101.04	$39.90	$121.74	$84.08	$210.72	$77.57
2012	$107.53	$44.74	$127.35	$87.47	$222.27	$73.55
2013	$110.82	$46.67	$130.91	$95.85	$227.08	$70.86
2014	$114.82	$48.29	$138.97	$157.14	$234.78	$68.66
2015	$119.16	$47.59	$146.60	$117.35	$238.39	$68.60

### Revenue

When the average total revenue per dog per year for all the years was calculated, dogs in the IM group and MHW groups generated up to $AU231.15 (US$166.43) per year for the clinic more than those in the reference group. The additional revenue was not just from the purchase of IM or MHW products but also from additional products and services (Table 4).

**Table 4:** Comparison of total annual revenue ($AU) per dog for dogs in reference group, IM group, or MHW group.

**Year**	**Reference group total revenue**	**Range**	**IM group total revenue**	**Range**	**IM group–revenue division**		**MHW group total revenue**	**Range**	**MHW group–revenue division**	
					**Revenue from IM only**	**Additional revenue**			**Revenue from MHW only**	**Additional revenue**
2010	$408.10	$402.59–$413.60	$429.14	$419.75–$438.53	$92.72	$336.42	$639.25	$617.03–$661.48	$129.52	$509.81
2011	$377.00	$372.36–$381.64	$459.47	$452.21–$466.73	$101.04	$358.43	$568.69	$553.25–$584.13	$121.74	$447.03
2012	$390.35	$386.04–$394.66	$502.02	$493.85–$510.19	$107.53	$394.49	$588.54	$569.70–$607.39	$127.35	$461.29
2013	$414.82	$408.99–$420.64	$519.85	$512.08–$527.63	$110.82	$409.03	$620.77	$600.38–$641.16	$130.91	$490.00
2014	$435.73	$430.96–$440.49	$557.34	$548.60–$566.08	$114.82	$442.52	$646.55	$628.05–$665.06	$138.97	$507.85
2015	$472.98	$468.24–$477.73	$552.60	$543.41–$561.79	$119.16	$433.44	$652.77	$632.84–$672.69	$146.60	$506.40

*IM, injectable moxidectin; MHW, monthly heartworm*.

Dogs in the IM group generated the most revenue per visit. When the revenue per visit was calculated, owners of dogs receiving two or more injections of moxidectin spent an average of $AU140.46 (US$101.13) per visit, compared to $AU119.40 (US$85.97) for those who purchased MHW prevention at least twice and $AU136.57 (US$98.33) for dogs in the reference group.

Dogs in the IM and MHW groups had more transactions with the practice overall than those that were in the reference group. Dogs in the IM group had an average of 3.6 transactions with their veterinarian per year compared to 3.1 for dogs in the reference group. Dogs in the MHW group had an average of 5.2 transactions per year.

### Retention

**Figure 5:**
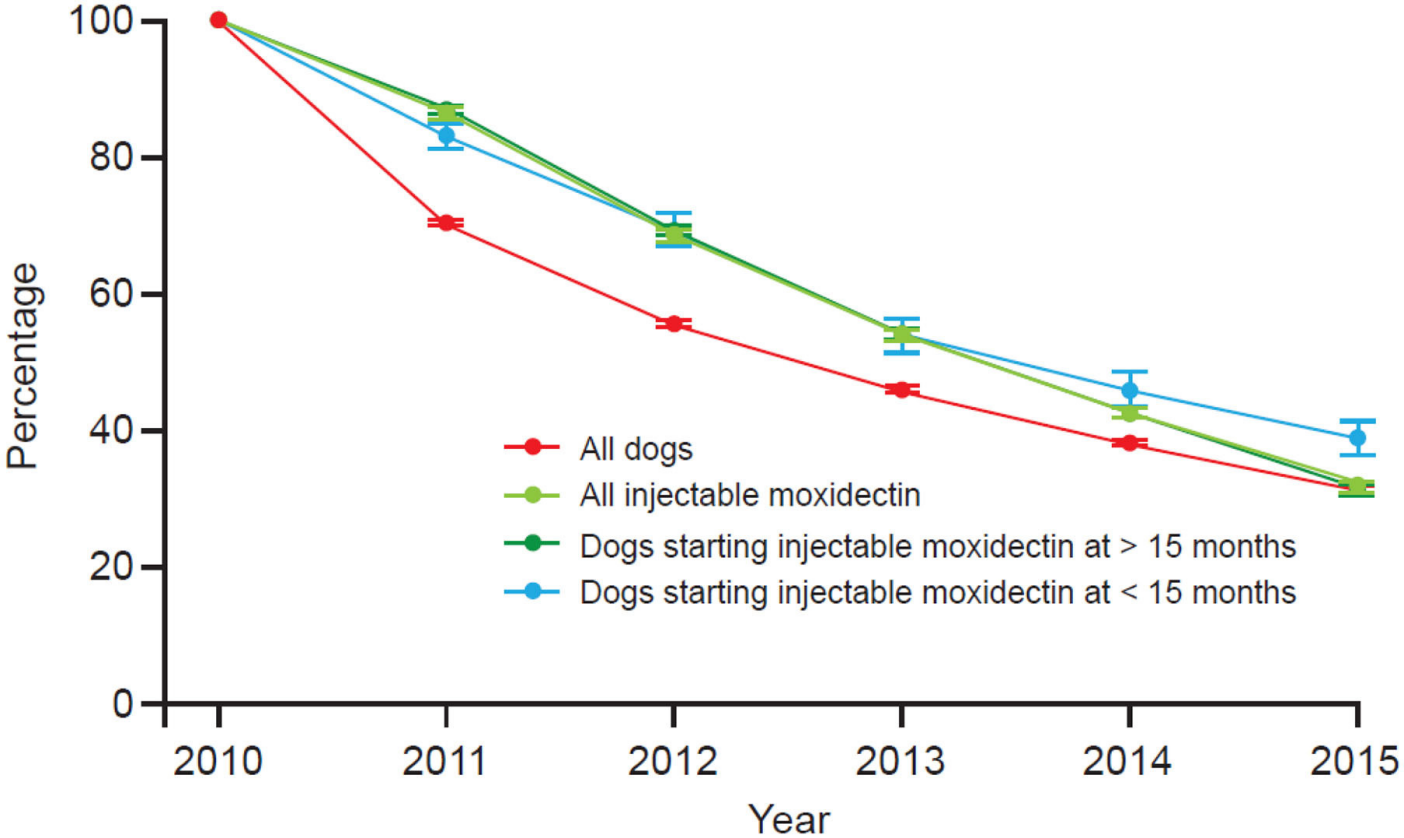
Retention plot for IM dogs vs. all dogs 2010–2015. Dogs < 15 months old are > 12 weeks old.

Dogs receiving IM, especially those that started at <15 months old, had a higher retention rate compared to the overall population of dogs, independent of the year of initiation (Figure 5). The average retention rate was the lowest among dogs in the reference group for all the years of study.

## Discussion

This study examined the economic value of HW preventative purchase compliance to the veterinary practice. When the average invoice per dog per year was calculated, dogs in the IM group or the MHW group generated more revenue for the veterinary practice per dog per year compared to dogs not repeatedly purchasing an HW preventative at the practice. The additional revenue came not only from the purchase of IM or MHW products but also from other products and services. Dogs in either prevention group also had more transactions with the practice than those in the reference group. With regard to the cost of prevention for owners, those buying MHW products spent more ($AU131.96/dog/year) than those buying IM ($AU108.29/dog/year), yet only 28.7% of dogs recorded sufficient MHW purchased to be single-year compliant, and only 24.4% of dogs prescribed MHW products purchased enough doses from their current veterinarian to provide protection for two consecutive years. Thus, there is clearly a societal and economic incentive for veterinary professionals to proactively communicate the disease risks and benefits of preventive medications to clients. Clients purchasing IM, especially those starting when their dogs were <15 months old, also had a higher clinic retention rate (i.e., stayed active with the practice longer) than the overall population of dogs, independent of the year of initiation.

The current study showed that during the period 2010–2015, the majority of dog owners, 73.0%, did not purchase HW preventatives repeatedly, i.e., more than once, from their veterinarian. This is important because an analysis of over 11 million veterinary medical records from the US revealed that dogs receiving an HW preventive were significantly less likely to develop HW disease compared to untreated dogs (25). Therefore, the provision of any method of HW prevention, not only IM, is important to reduce HW disease. It has been demonstrated that owner lack of compliance with instructions for monthly administration occurs frequently and is believed to be the most common explanation for prevention failures associated with oral MHW products in particular (24). It has also been recommended that consistent use of existing, effective HW preventives should be the primary goal in reducing the prevalence of HW infection (26).

In this study, we examined two types of compliance: single-year purchase compliance (SYPC) and consecutive-year purchase compliance (CYPC). For each single year in the observation period, purchase compliance (receipt of IM in the course of the year, regardless of the interval between injections between years, but with dogs under 15 months of age requiring at least two doses since birth to be classified as compliant) with IM, which lasts up to 12 months, ranged from 92.8% to 96.9% compared with only 27.0–36.5% for dogs receiving MHW products, which requires sufficient purchases in the year to cover 12 monthly doses, or an equal number of doses for a dog <12 months old as its age in months at the end of the year. The reason that SYPC with IM was not 100% is likely a result of dogs <15 months not getting the stipulated minimum of two injections since birth.

It was also important in this pharmacoeconomics study to establish the proportion of dog owners purchasing 12 months' supply of MHW preventatives who continued to do so in consecutive years and provide a similar comparison for IM. This was done by measuring consecutive-year purchase compliance (CYPC), i.e., either receipt of IM in the current and previous years or enough purchases of MHW preventatives to cover 12 monthly doses in the current and previous years. CYPC for MHW preventives was only 24.4% (95% CI = 24.0–24.8%), and so only 24.4% of these owners were purchasing enough products through their veterinarian to provide continuous protection over the observation period. In contrast, CYPC with IM was 76.7% (95% CI = 76.5–76.9%), and so 76.7% of dogs receiving a moxidectin injection were protected over the observation period.

In Australia, pet owners are able to source MHW preventives outside the veterinary channel. This is reflected in our data, where 73% of dogs were owned by pet owners who did not purchase HW preventives consistently from their veterinarian. The low MHW prevention purchase compliance identified in our study must be viewed through the context of overall MHW preventive purchases in Australia. There are likely different attitudes of owners and veterinarians to prevention and routes of administration, which factor into choosing an appropriate HW preventive. For pet owners, purchasing preventives outside of the veterinary channel is potentially cheaper. However, as previously stated, veterinarians are unable to track purchase compliance with such products or determine if they have been administered as required.

One retrospective review of US veterinary clinic medical records involving cases of suspected lack of effectiveness for an HW preventive found that in 80.7% of cases, there was insufficient HW preventive purchased, with purchase gaps of >45 days, thus providing a potential window of infection (13). Another retrospective study of dog records in a US veterinary teaching hospital examined factors associated with preventative use and areas of potential weakness in client communication (27). How well owners comply with, or adhere to, treatment recommendations made by veterinary practitioners can significantly affect the success of medications and subsequent health outcomes (28). In one study, it was found that overall, only 13–23% of patients were questioned about HW, flea, or tick preventative use during routine medical history taking (27). MHW preventive purchase through the veterinarian is potentially associated with a physical examination to ensure that the dog is in good health and a good candidate for the preventative; however, IM administration is always associated with a veterinary examination. Bringing the dog to the clinic for either type of HW preventive offers additional opportunities to examine the dog and ask questions about preventive care. Moreover, continued engagement with pet owners remains fundamental in reinforcing the importance of HW prevention. It is also an avenue for the owner to voice any concerns about their dog's health and ultimately identify new health problems that may have developed.

Our study examined the average age and weight profiles of dogs in the IM, MHP, and reference groups and found no differences. These data indicate that dog owners do not discriminate between HW prevention choices based on age or weight, with the likelihood of purchase of HW products from their veterinary clinic being influenced by other factors not identified by this study.

The current study utilized transaction records for the period 2010–2015 of products and services purchased by dog-owning veterinary clients. This was a large sample spanning 52 veterinary clinics covering all Australian states and evaluating the records of 228,185 dogs. However, examination of clinic transaction records to monitor pet owner medication use has important limitations. Purchase history does not equate to the number of medication doses administered to a pet, nor does it capture purchases made outside the veterinary clinic (22). Another limitation of the current study was the fact that it used only descriptive statistics to analyze the results. Other potential limitations of the study include the fact that the compliance definition was based on full year coverage and does not consider any seasonality or regionality of HW risk. Nevertheless, 12 months of coverage is consistent with the recommendations of both AHS and the Australian Heartworm Advisory Panel guidelines.

The financial data are from Australia; therefore, caution should be exercised when generalizing the revenue results more widely. Furthermore, transactional data were reported as nominal figures and no adjustments were made for inflation, and no data were included on acquisition costs and price changes from year to year and thus do not accurately capture financial benefit. Transactional data were also not corroborated with medical data; therefore, potential clinical confounders may have been missed. Finally, data for MHW purchases were attributed to individual dogs. There remains a potential for dose sharing in multidog households, which has been cited as a factor for the incidence of HW disease (13) but could not be accounted for in this study. Importantly, the focus of this study was HW preventives sourced from the veterinarian, thus capturing only the veterinary perspective. Ideally, national data on non-veterinary clinic transactions for monthly preventive options would provide a more robust understanding of purchase compliance from a pet owner perspective. This was beyond the scope of this study and is a potential avenue for future research.

## Conclusion

This study of veterinary practices in Australia showed that 73% of 228,185 dog owners did not purchase HW preventatives more than once from their veterinarian during the study period (2010–2015). Of those owners who did purchase HW preventative medication more than once, 76.7% of dogs receiving IM were protected for at least 24 consecutive months during the entire 5-year period, whereas only 24.4% of dog owners on monthly HW prevention were purchasing enough products from the veterinary practice in the study to provide 24 consecutive months of protection. Also, although repeat monthly HW purchasers only purchased enough products to cover an average of 7.2 months of each year between 2010 and 2015, they spent more than IM owners per year on HW prevention at the veterinary practice (average annual spend of A$108.29/dog/year on IM compared to $131.96/dog/year on monthly HW prevention). Finally, dogs receiving repeated IM or monthly HW prevention generated more revenue for the veterinary practice per dog per year compared to dogs not repeatedly purchasing any HW preventative at the practice. IM generated the most revenue/visit compared with both other groups.

## Data Availability Statement

The raw data supporting the conclusions of this article will be made available by the authors, without undue reservation.

## Author Contributions

AW was responsible for study design, analysis, and interpretation. KG was responsible for analysis, production of descriptive summaries, and data visualization. KM was responsible for analysis, interpretation, and write-up of data. T-SN was responsible for study design. RL'E was responsible for data quality assurance and manuscript finalization. All authors read and approved the final manuscript.

## Conflict of Interest

KG was employed by Athens Technology Center. KM, AW, RL'E, and T-SN are employed by Zoetis Inc. The authors declare that this study received funding from Zoetis. The funder had the following involvement with the study: study design, analysis, interpretation, write up of data, data quality assurance and manuscript finalization.

## Abbreviations

AHS: American Heartworm Society
CI: confidence interval
CYPC: consecutive-year purchase compliance
HW: heartworm
IM: injectable moxidectin
MWH: monthly heartworm
SYPC: single-year purchase compliance
SD: standard deviation
US: United States.

## References

[1] Lee ACY Montgomery SP Theis JH Blagburn BL Eberhard ML. Public health issues concerning the widespread distribution of canine heartworm disease. Trends Parasitol. (2010) 26:168–73. 10.1016/j.pt.2010.01.003 20181530

[2] American Heartworm Society. Current Canine Guidelines for the Prevention, Diagnosis, and Management of Heartworm (Dirofilaria immitis) Infection in Dogs. (2018). Available online at: https://www.heartwormsociety.org/veterinary-resources/american-heartworm-society-guidelines (accessed April 2019).

[3] Morchón R Carretón E González-Miguel J Mellado-Hernández I. Heartworm disease (*Dirofilaria immitis*) and their vectors in Europe – new distribution trends. Front Physiol. (2012) 3:196. 10.3389/fphys.2012.00196 22701433 PMC3372948

[4] Nguyen C Koh WL Casteriano A Beijerink N Godfrey C Brown G. Mosquito-borne heartworm *Dirofilaria immitis* in dogs from Australia. Parasit Vectors. (2016) 9:535. 10.1186/s13071-016-1821-x 27717406 PMC5055658

[5] US Food and Drug Administration. Keep the Worms Out of Your Pet's Heart! The Facts about Heartworm Disease. (2017). Available online at: https://wayback.archive-it.org/7993/20171104084459/https://www.fda.gov/AnimalVeterinary/ResourcesforYou/AnimalHealthLiteracy/ucm188470.htm (accessed 24 April 2019).

[6] Brown H Harrington LC Kaufman PE McKay T Bowman DD Nelson CT. Key factors influencing canine heartworm, *Dirofilaria immitis*, in the United States. Parasit Vectors. (2012) 5:245. 10.1186/1756-3305-5-245 23111089 PMC3523980

[7] American Heartworm Society. AHS Announces Findings of New Heartworm Incidence Survey. (2017). Available online at: https://heartwormsociety.org/newsroom/in-the-news/347-ahs-announces-findings-of-new-heartworm-incidence-survey (accessed July 26, 2017).

[8] Genchi C Bowman D Drake J. Canine heartworm disease (*Dirofilaria immitis*) in Western Europe: survey of veterinary awareness and perceptions. Parasit Vectors. (2014) 7:206. 10.1186/1756-3305-7-206 24779376 PMC4013803

[9] Bowman DD Atkins CE. Heartworm biology, treatment, and control. Vet Clin North Am Small Anim Pract. (2009) 39:1127–58. 10.1016/j.cvsm.2009.06.003 19932367

[10] European Society of Dirofilariosis and Angiostrongylosis. Guidelines for Clinical Management of Canine Heartworm Disease. (2017). Available online at: https://www.esda.vet/wp-content/uploads/2017/11/GUIDELINES-FOR-CLINICAL-MANAGEMENT-OF-CANINE-HEARTWORM-DISEASE.pdf (accessed April 23, 2019).

[11] Zoetis Australia Pty Ltd. Proheart SR-12 Injection Once-A-Year Heartworm Preventative for Dogs. Proheart SR12 Label (2015).

[12] Australian Heartworm Advisory Panel. Australian Guidelines for Heartworm Prevention in Dogs. (2014).

[13] Atkins CE Murray MJ Olavessen LJ Burton KW Marshall JW Brooks CC. Heartworm ‘lack of effectiveness’ claims in the Mississippi delta: computerized analysis of owner compliance −2004–2011. Vet Parasitol. (2014) 206:106–13. 10.1016/j.vetpar.2014.08.013 25440944

[14] Bowman D. Heartworms, macrocyclic lactones, and the specter of resistance to prevention in the United States. Parasit Vectors. (2012) 5:138. 10.1186/1756-3305-5-138 22776618 PMC3406940

[15] American Heartworm Society. Heartworm Incidence Maps. (2018). Available online at: https://www.heartwormsociety.org/veterinary-resources/incidence-maps (accessed December 10, 2020).

[16] Companion Animal Parasite Council. Available online at: https://capcvet.org/maps/#2019/5/heartworm-canine/dog/united-states/ (accessed December 10, 2020).

[17] US Environmental Protection Agency. Joint Statement on Mosquito Control in the United States. Available online at: https://www.epa.gov/mosquitocontrol/joint-statement-mosquito-control-united-states (accessed July 2019).

[18] Heartworm Surveillance Map. Available online at: www.vetsaustralia.com.au (accessed October 2019).

[19] Vetwest Animal Hospitals. Heartworm Disease. (2019). Available online at: https://www.vetwest.com.au/proheart-sr12-injection (accessed April 22, 2019).

[20] Drake J Wiseman S. Increasing incidence of *Dirofilaria immitis* in dogs in USA with focus on the southeast region 2013–2016. Parasit Vectors. (2018) 11:39. 10.1186/s13071-018-2631-0 29343304 PMC5773159

[21] American Pet Products Association. 2017–2018 APPA National Pet Owners Survey. Greenwich. Available online at: http://www.americanpetproducts.org (accessed June 18, 2018).

[22] Lavan R Heaney K Vaduvoor SR Tunceli K. A comparative analysis of heartworm medication use patterns for dogs that also receive ectoparasiticides. Parasit Vectors. (2018) 11:493. 10.1186/s13071-018-3076-1 30170612 PMC6119298

[23] Peterson AM Nau DP Cramer JA Benner J Gwadry-Sridhar F Nichol M. A checklist for medication compliance and persistence studies using retrospective databases. Value Health. (2007) 10:3–12. 10.1111/j.1524-4733.2006.00139.x 17261111

[24] Australian Veterinary Association Ltd. Australian Veterinary Workforce Survey. (2016). Available online at: https://www.ava.com.au/sites/default/files/AVA_website/pdf/AVA-Workforce-Survey-2016-Final.pdf (accessed April 2017).

[25] Glickman LT Glickman NW Moore GE Lok JB McCall JW Lewis HB. Comparative effectiveness of sustained-release moxidectin (ProHeart 6) and ivermectin (Heartgard Plus) for the prevention of heartworm infection in dogs in the United States. Intern J Appl Res Vet Med. (2006) 4:339–54.

[26] Ku TN. Investigating management choices for canine heartworm disease in northern Mississippi. Parasit Vectors. (2017) 10:474. 10.1186/s13071-017-2450-8 29143688 PMC5688398

[27] Gates MC Nolan TJ. Factors influencing heartworm, flea, and tick preventative use in patients presenting to a veterinary teaching hospital. Prev Vet Med. (2010) 93:193–200. 10.1016/j.prevetmed.2009.10.012 19931925 PMC2815201

[28] Wareham KJ Brennan ML Dean RS. Systematic review of the factors affecting cat and dog owner compliance with pharmaceutical treatment recommendations. Vet Rec. (2019) 184:154. 10.1136/vr.104793 30455188

