# Corrigendum: Retrospective Analysis of Heartworm (*Dirofilia immitis*) Prevention Medication Compliance and Economic Value in Dogs in Veterinary Practices in Australia

**DOI:** 10.3389/fvets.2021.759537

**Published:** 2021-10-08

**Authors:** Kennedy Mwacalimba, Andrea Wright, Konstantinos Giannakakis, Richard L'Estrange, Tinh-Son Nguyen

**Affiliations:** ^1^Outcomes Research, Zoetis, Parsippany, NJ, United States; ^2^Athens Technology Center S.A., Halandri, Greece; ^3^Zoetis Australia, Silverwater, NSW, Australia

**Keywords:** heartworm, dog, extended-release, injectable moxidectin, revenue, *Dirofilaria immitis*

In the original article, there was an error. The funding statement declares that the funder was not involved in the study design, collection, analysis, interpretation of data, the writing of this article or the decision to submit it for publication.

A correction has been made to the **Funding** statement:

The authors declare that this study received funding from Zoetis.

A correction has been made to the **Conflict of Interest** statement:

“KG was employed by Athens Technology Center. KM, AW, RL'E, and T-SN are employed by Zoetis Inc.

The authors declare that this study received funding from Zoetis. The funder had the following involvement with the study: study design, analysis, interpretation, write up of data, data quality assurance and manuscript finalization.”

The authors apologize for this error and state that this does not change the scientific conclusions of the article in any way. The original article has been updated.

## Publisher's Note

All claims expressed in this article are solely those of the authors and do not necessarily represent those of their affiliated organizations, or those of the publisher, the editors and the reviewers. Any product that may be evaluated in this article, or claim that may be made by its manufacturer, is not guaranteed or endorsed by the publisher.

